# Mutation analysis of disease causing genes in patients with early onset or familial forms of Alzheimer’s disease and frontotemporal dementia

**DOI:** 10.1186/s12864-022-08343-9

**Published:** 2022-02-04

**Authors:** María Pagnon de la Vega, Carl Näslund, RoseMarie Brundin, Lars Lannfelt, Malin Löwenmark, Lena Kilander, Martin Ingelsson, Vilmantas Giedraitis

**Affiliations:** 1 Department of Public Health and Caring Sciences/Geriatrics, Uppsala University, Uppsala, Sweden; 2 Krembil Brain Institute, University Health Network, Toronto, Canada; 3 Department of Medicine and Tanz Centre for Research in Neurodegenerative Diseases, University of Toronto, Toronto, Canada

**Keywords:** Alzheimer's disease, Frontotemporal dementia, Neurodegenerative disorders, *PSEN1*, *PSEN2*, *APP*, *MAPT*, *APOE*, Exome sequencing

## Abstract

**Background:**

Most dementia disorders have a clear genetic background and a number of disease genes have been identified. Mutations in the tau gene (*MAPT*) lead to frontotemporal dementia (FTD), whereas mutations in the genes for the amyloid-β precursor protein (*APP*) and the presenilins (*PSEN1, PSEN2*) cause early-onset, dominantly inherited forms of Alzheimer’s disease (AD).

Even if mutations causing Mendelian forms of these diseases are uncommon, elucidation of the pathogenic effects of such mutations have proven important for understanding the pathogenic processes. Here, we performed a screen to identify novel pathogenic mutations in known disease genes among patients undergoing dementia investigation.

**Results:**

Using targeted exome sequencing we have screened all coding exons in eleven known dementia genes (*PSEN1*, *PSEN2*, *APP*, *MAPT*, *APOE*, *GRN*, *TARDBP*, *CHMP2B*, *TREM2*, *VCP* and *FUS*) in 102 patients with AD, FTD, other dementia diagnoses or mild cognitive impairment.

We found three AD patients with two previously identified pathogenic mutations in *PSEN1* (Pro264Leu and Met146Val). In this screen, we also identified the recently reported *APP* mutation in two siblings with AD. This mutation, named the *Uppsala mutation*, consists of a six amino acid intra-amyloid β deletion.

In addition, we found several potentially pathogenic mutations in *PSEN2*, *FUS, MAPT, GRN* and *APOE*. Finally, *APOE ε*4 was prevalent in this patient group with an allele frequency of 54%.

**Conclusions:**

Among the 102 screened patients, we found two disease causing mutations in *PSEN1* and one in *APP*, as well as several potentially pathogenic mutations in other genes related to neurodegenerative disorders. Apart from giving important information to the clinical investigation, the identification of disease mutations can contribute to an increased understanding of disease mechanisms.

## Introduction

Mutations in the genes for the amyloid-β precursor protein (*APP*) and the presenilins (*PSEN1* and *PSEN2*) cause early-onset, dominantly inherited forms of Alzheimer’s disease (AD), whereas mutations in the *MAPT* gene mainly lead to frontotemporal dementia (FTD). The elucidation of the pathogenic effects of such mutations has proven important for the understanding of the respective disease processes.

Alzheimer’s disease is neuropathologically characterized by the formation and extracellular deposition of insoluble plaques, consisting of the 38–43 amino acid long peptide amyloid-β (Aβ) and intracellular neurofibrillary tangles of the tau protein, as well as a substantial neuronal loss [1]. According to the amyloid cascade hypothesis, AD is initiated by increased levels or a changed conformation of Aβ which can promote the formation of toxic oligomers and protofibrils before the formation of insoluble plaques [2]. We have previously identified and characterized two different *APP* mutations, the *Swedish* mutation [3] and the *Arctic* mutation [4]. Functional analyses of these mutations have significantly increased our understanding of the disease pathogenesis. Whereas the *Swedish* mutation results in an increased cleavage by β-secretase and thereby elevated levels of all forms of Aβ [5, 6], the *Arctic* mutation leads to a conformational change of Aβ and increased formation of toxic Aβ protofibrils [7]. Moreover, mutations in *PSEN1* and *PSEN2* have been shown to increase the generation of the longer and more aggregation prone form of Aβ (Aβ42).

Recently, several new genes causing dementia disorders have been identified. Such discoveries have been important to increase our knowledge of the aetiology behind these disorders. Most notably, the understanding of frontotemporal dementia has been further advanced by the identification of disease causing mutations in the genes for TAR DNA-binding protein-43 (*TARDBP*), progranulin (*GRN*) and C9orf72-SMCR8 complex subunit (*C9ORF72*) [8–12]. Moreover, discoveries of rare variants in the triggering receptor expressed on myeloid cells 2 (*TREM2*) gene have highlighted the involvement of immunological and inflammatory pathways in AD pathogenesis [13, 14].

The development of next generation sequencing technologies has enabled identification of new mutations in previously known as well as in novel disease genes [15]. Furthermore, there is growing evidence that mutation screening may yield interesting findings also in patients with late onset disease forms [16].

In this study we have performed exome sequencing of eleven potential disease genes (*PSEN1*, *PSEN2*, *APP*, *MAPT*, *APOE*, *GRN*, *TARDBP*, *CHMP2B*, *TREM2*, *VCP* and *FUS*) in patients with AD and other neurodegenerative diseases for whom an early disease onset and/or a familial pattern of inheritance had been reported.

## Results

Three different disease causing mutations and several mutations with potential pathogenicity were identified in the screened gene regions (Table 1).

**Table 1:** Clinical characteristics of patients with known or potentially pathogenic mutations

Patient No	Diagnosis	Gender	Onset age	Heredity	Protein change (dbSNP)	APOE	Function	Pathogenic	Population frequency^a^	SIFT score^b^	PolyPhen-2 score^b^
1	AD	F	50	Yes	PSEN1 P264L (rs63750301)	ε3/ε4	Missense	Yes	0.0000040	0	1
2	AD	M	42	Yes	PSEN1 P264L (rs63750301)	ε3/ε4	Missense	Yes	0.0000040	0	1
3	AD	M	34	No	PSEN1 M146V (rs63750306)	ε3/ε3	Missense	Yes	-	0.01	0.985
4	AD	M	65	Yes	PSEN2 I144L (rs764718172)	ε3/ε3	Missense	Unknown	0.0000080	0.01	0.257
5	bvFTD	M	69	Yes	PSEN2 A252T (rs138836272)	ε3/ε3	Missense	Unknown	0.00023	0.42	0.172
					FUS Δ229-231 (rs767564995)		Deletion	Unknown	0.00021	-	-
6	AD	M	59	Yes	FUS Ser57Δ (rs777545405)	ε4/ε4	Deletion	Unknown	0.00017	-	-
7	svPPA	M	64	Yes	FUS Δ166-167 (rs537605135)	ε4/ε4	Deletion	Unknown	0.00030	-	-
8	AD	F	57	Yes	GRN A324V (rs758636128)	ε3/ε3	Missense	Unknown	0.000024	0.21	0.129
9	AD	M	59	Yes	MAPT V224G (rs141120474)	ε4/ε4	Missense	Unknown	0.0021	0	0.187
10	MCI	M	61	Yes	MAPT A239T (rs63750096)	ε3/ε4	Missense	Unknown	0.00063	0.27	0.079
11	AD	M	56	Yes	APOE R269G (rs267606661)	ε3/ε4	Missense	Unknown	0.00036	0	0.533
12	AD	M	44	Yes	APP Δ690-695	ε3/ε3	Deletion	Yes	-	-	-
13	AD	M	40	Yes	APP Δ690-695	ε3/ε3	Deletion	Yes	-	-	-

^a^Population frequency was obtained from gnomAD database, except for rs767564995 for which dbSNP was used

^b^PolyPhen-2 and SIFT scores were obtained using Ensembl Variant Effect Predictor. These scores are used to predict whether amino acid substitution is likely to affect protein function. The SIFT score ranges from 0.0 (deleterious) to 1.0 (tolerated). The PolyPhen-2 score ranges from 0.0 (tolerated) to 1.0 (deleterious)

**Fig. 1:**
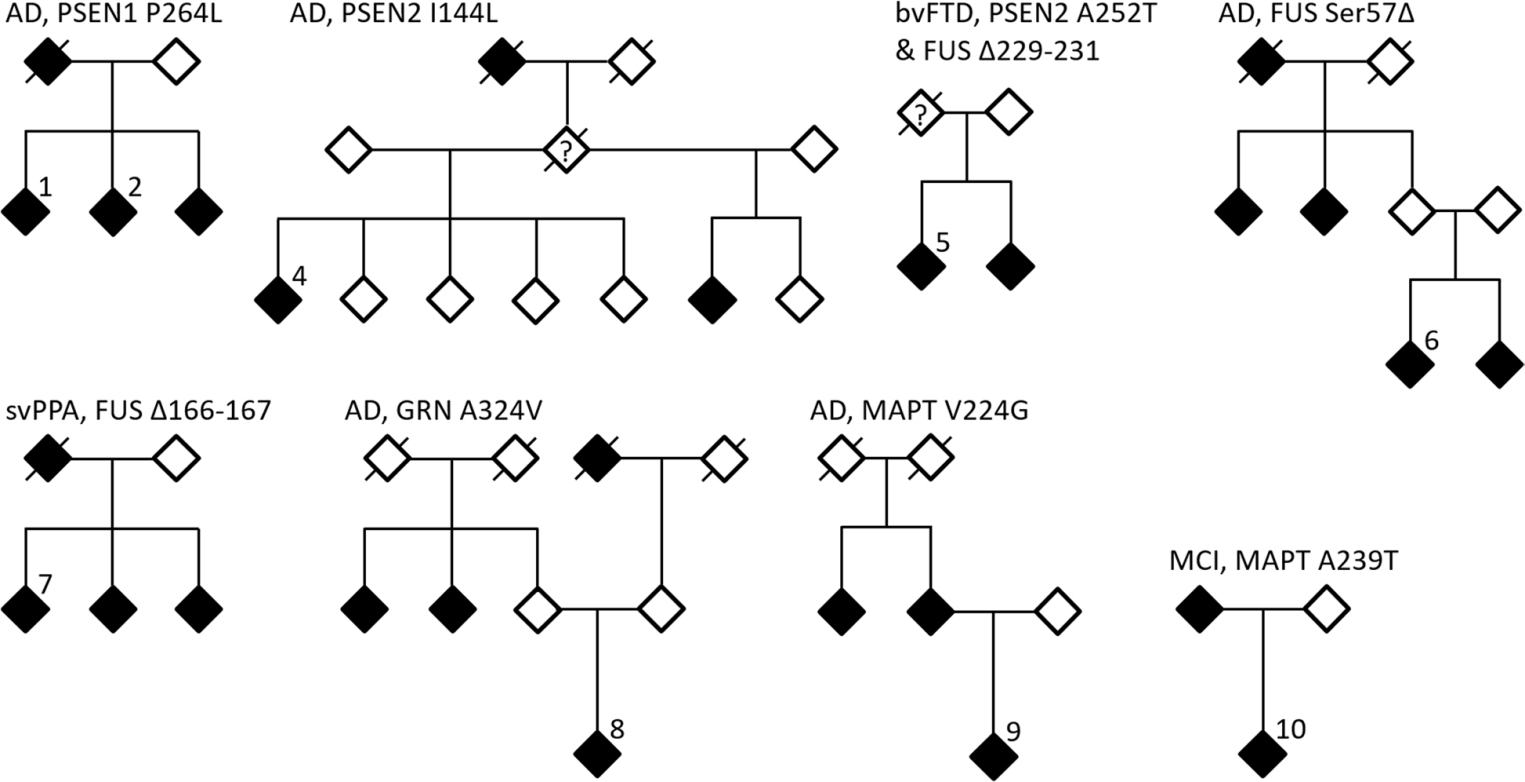
Pedigrees of the families in which mutations were identified. The analyzed cases are indicated by numbers. Each pedigee is labeled by diagnosis and the nature of the mutation(s) identified. Filled symbols are affected family members. Slashed symbols are used for individuals known to be deceased. The numbers refer to the screened patients, corresponding to Table 1

We found two pathogenic mutations in the *PSEN1* gene, both of which had been previously reported [17–19]. The *PSEN1* P264L (rs63750301) mutation was detected in two siblings with AD. In the same family, the father and another sibling also suffered from the disease, although DNA samples were not available from them (Fig. 1). The age at onset in this family was reported to be 40–50 years. The siblings suffered from global cognitive impairment and early personality changes and both displayed a clear hypometabolism in posterior temporo-parietal cortex on FDG-PET. The other *PSEN1* mutation, M146V (rs63750306), was identified in an AD patient who had presented with slight short term memory dysfunction and spatial disorientation already at about 30 years of age. At later disease stages the patient suffered from myoclonic epileptic seizures and gait difficulties. A widespread bilateral parietal hypometabolism with involvement also of temporal and frontal cortices was observed on FDG-PET. Interestingly, no heredity had been reported in this family.

In the Aβ coding sequence of the *APP* gene we identified the recently reported eighteen base pair deletion, which causes a six amino acid deletion in the protein sequence. This in frame deletion, named the *Uppsala mutation*, was found in two siblings and a cousin suffering from an aggressive early onset form of AD. A typical pattern of autosomal dominant inheritance and age at disease onset of 40–50 years had been reported in earlier generations of the family [20]. We have also analyzed more than 500 DNA samples from Swedish AD patients, older family members, and older healthy control subjects, without finding any other cases with this mutation. Furthermore, we extensively studied clinical and pathological features of this deletion and the data strongly suggest that the *Uppsala mutation* is the cause of AD in this family [20].

In addition to these pathogenic mutations, several mutations with potential pathogenicity were identified. In one individual with AD and a known family history of dementia we found a Ser57 deletion (rs777545405) in *FUS* (Fig. 1). This deletion had been previously reported as likely pathogenic in a patient with sporadic amyotrophic lateral sclerosis (ALS) [21]. Our patient had experienced memory problems before the age of 60 years, but no typical ALS symptoms had been reported. In addition, two other deletions in *FUS* were found in patients with behavioral variant FTD (bvFTD) and semantic variant primary progressive aphasia (svPPA), respectively (Table 1).

In *PSEN2*, the A252T mutation (rs138836272) was identified in a patient with the behavioral variant of FTD (bvFTD), also carrying the Δ229-231 deletion in *FUS* (Fig. 1 and Table 1). The *PSEN2* A252T mutation had been previously reported in African healthy controls [22] and its pathogenicity is thus unclear. In addition, *PSEN2* I144L (rs764718172), which has not been reported in any previous studies, was found in an AD patient with a family history of disease (Fig. 1).

Since *APOE* was included in our next generation sequencing analysis, we examined all *APOE* exons for possible mutations. In one patient with early onset AD we identified the R269G (rs267606661) mutation. This *APOE* mutation had previously been reported as a likely cause of autosomal dominant hypercholesterolemia [23].

Moreover, three rare genetic variants with potential pathogenicity were found in *MAPT* and *GRN* (Fig. 1 and Table 1), whereas no variants that are likely to be pathogenic could be identified in *TARDBP*, *CHMP2B* and *VCP*.

Analyses of conventional *APOE* alleles in the total sample set showed an *ε*4 allele frequency of 54%. Of the 102 patients, 32 were homozygous for *ε*4 and 45 had the *ε*3*ε*4 genotype. When only including the 77 AD cases in the analysis, a slightly higher *APOE ε*4 allele frequency was observed (57%) (Table 2).

**Table 2:** Description of the study population

Disease	Number of patients, n	Known heredity, n (%)	Females, n (%)	Age at onset, mean (range)
Alzheimer’s disease	77	70 (90.9)	36 (46.8)	60.8 (34–76)
Behavioral variant of frontotemporal dementia	7	7 (100)	2 (28.6)	59.1 (47–69)
Other types of frontotemporal dementia	4	3 (75)	1 (25)	60.8 (54–71)
Semantic variant primary progressive aphasia	5	4 (80)	2 (40)	58,2 (49–64)
Other dementia related diagnoses^a^	2	1 (50.0)	0 (0)	65.0 (55–75)
Mild cognitive impairment^b^	7	7 (100)	4 (57.1)	59.4 (55–65)

^a^In this group one patient had vascular dementia and one unspecified dementia

^b^Most patients with MCI had a known heredity for AD

We also included *TREM2* in our analyses, since several studies have recently reported that rare variants in this gene could confer an increased AD risk similar to *APOE ε*4 [13, 24]. We found that eight AD and two patients with mild cognitive impairment (MCI) were carriers of the *TREM2* R62H allele (rs143332484), which gives an allele frequency of 4.9%. This is significantly higher than what has been previously reported in European populations [25]. Interestingly, also the two AD patients with the *Uppsala mutation* were found to carry the *TREM2* R62H allele.

## Discussion

In this study, we have screened 102 patients with AD or FTD from our Memory clinic and identified several pathogenic mutations causing AD. We also found two *PSEN1* mutations, P264L and M146V. Both of these mutations have been previously described as pathogenic, causing autosomal dominantly inherited disease forms [17–19]. The approximate onset age of *PSEN1* P264L patients had been reported at 45 years, which corresponds well to the onset ages of our patients. Despite the fact that all individuals with this mutation get dementia at an early age, there is an interindividual variation of symptoms. In the study by Martikainen et al., one of the three patients carrying this mutation displayed spastic paraparesis, whereas the other two patients presented with parkinsonism. With respect to neuropathology, all three cases exhibited abundant cotton wool plaques in the brain at autopsy [18].

The *PSEN1* M146V mutation usually causes early onset AD with an onset age at about 40 years [26]. Two mutation-carrying patients from a previously described large Swedish/Finnish family showed global cortical glucose hypometabolism, which was further accentuated over time. Brain examination revealed considerably higher numbers of neuritic plaques and neurofibrillary tangles in all examined brain regions, as compared to sporadic AD patients. Similar to our patient, epileptic seizures were observed in both patients described in this study [27].

The recently reported deletion in *APP*, resulting in a six amino acid shorter version of the Aβ peptide, is to our knowledge the first dominantly inherited form of AD caused by a large deletion in Aβ [20﻿]. Previously, only a single amino acid deletion within the Aβ sequence has been reported in a Japanese family. This *APP* mutation was described to have a recessive mode of inheritance and seems to cause disease due to an increased oligomerization and higher resistance to proteolytic degradation of Aβ [28]. As recently described, the *Uppsala APP* mutation causes increased Aβ generation by altering α-secretase and β-secretase cleavages of APP. Furthermore, the Uppsala Aβ mutant adopts unique polymorphs that accelerate the formation of fibrils and their deposition into amyloid plaques [20].

The Ser57 deletion in *FUS* and the R269G mutation in *APOE* have been previously reported, although clinical data from patients and data about functional consequences of these mutations are still limited. Nevertheless, the clinical presentation of the reported cases seems to be very different from our patients [21, 23].

Several other mutations identified in our study have been previously reported in genetic analyses of AD and other dementia disorders. The *MAPT* A239T variant was described in an FTD patient carrying pathogenic deletion in *GRN* gene [29]. The *MAPT* V224G was reported in several studies and found in both AD patients and controls [16, 30]. The *PSEN2 A252T* mutation, affecting an amino acid residue that is conserved between *PSEN1* and *PSEN2*, was found in two controls from the Mandenka and Yoruba samples [22]. Nevertheless, only very few individuals with these mutations have been reported and further analyses are necessary in order to examine their possible pathogenic role.

The *APOE ε4* allele is the strongest risk factor for late onset AD [31]. In the general Caucasian population *APOE ε4* frequency is around 10–15% whereas in AD a frequency of 25–35% is usually reported [32, 33]. Thus the high *APOE ε4* frequency of over 50% in the patients included in our study, confirms that *APOE* is of importance also in familial dementia [34–36]. Much more surprising was that the frequency of the *TREM2* R62H allele was as high as 4.9%, whereas the allele frequency reported in dbSNP for this variant was just about 1% in most European and American populations. In the first study investigating a potential association of *TREM2* R62H to AD, this polymorphism was not associated with disease [14]. However, in another study of 85 133 subjects, a strongly significant association between AD and *TREM2* R62H was found, although the allele frequency was low (1.4% in AD cases and 0.9% in controls) [25].

In this study we used a sequence enrichment technology together with next generation sequencing in order to screen target genes. An advantage of this method is its cost efficiency combined with the high quality of sequencing data. The main limitation of the technique is that only already known disease genes can be analyzed. Another limitation is that it cannot be used to analyze the structural variants, like deletions and insertions, and repetitive sequences like the hexanucleotide repeat expansions in *C9ORF72* causing FTD [11, 12]. Furthermore, a number of genes associated with increased susceptibility for AD have been identified [37, 38]. Variations in several of these genes, such as *CR1*, *SORL1*, *BACE1*, *ABCA7*, could also predispose for familial early onset form of the disease [39, 40]. Some of the families included in our study have rather complex patterns of inheritance (Fig. 1) and we can therefore not exclude that combination of variants in several genes can be disease causative. Nevertheless, *PSEN1*, *PSEN2* and *APP* mutations are the main causes of autosomal-dominant early-onset AD, whereas mutations in *MAPT* and *GRN* account for a significant part of genetic FTD [41–43].

In conclusion, in our screen of 102 AD, FTD and MCI patients with early onset and/or signs of heredity we found two previously described *PSEN1* mutations, the recently described *Uppsala APP* deletion and a number of other potentially pathogenic mutations. Screening for mutations in known and putative disease susceptibility genes can aid in the clinical diagnosis of dementia patients. Moreover, such screening could enable us to discover additional disease mechanisms that can be targeted by novel therapeutic strategies.

## Methods

### Selection of patients and controls

A selection of 102 patients who attended the Memory clinic at Uppsala University Hospital during 2006–2019 were recruited to the study. The included patients had been diagnosed with either AD, FTD, another dementia disorder or mild cognitive impairment. Moreover, they had displayed a disease onset before 65 years of age and/or had reported at least one first degree relative diagnosed with a dementia disorder. The diagnoses were based on established criteria including analysis of cerebrospinal fluid biomarkers of AD in most cases [44–48]. The patient information is summarized in Table 2. Available pedigrees for patients with identified mutations are presented in the Fig. 1. The pedigree for the patients with the *Uppsala mutation* was recently published [20].

### Gene selection

Nine genes with known pathogenic mutations causing familial early onset dementia diseases were included in the analyses (*APP*, *PSEN1*, *PSEN2*, *MAPT*, *GRN*, *TARDBP*, *CHMP2B*, *VCP* and *FUS*). In addition, we analyzed *TREM2* in which rare variants have been associated with an increased risk of AD [14, 25]. We also included *APOE* in order to determine the *ε*2-4 genotypes. The gene information is summarized in Table 3.

**Table 3:** Gene list

Gene name	Gene symbol	Chromosome	Associated disease
Presenilin 2	*PSEN2*	chr01	AD, FTD
TDP-43, TAR DNA binding protein	*TARDBP*	chr01	FTD, ALS
Charged multivesicular body protein 2B	*CHMP2B*	chr03	FTD, AD
Triggering receptor expressed on myeloid cells 2	*TREM2*	chr06	AD
Valosin containing protein	*VCP*	chr09	ALS, FTD
Presenilin 1	*PSEN1*	chr14	AD, FTD
FUS RNA binding protein	*FUS*	chr16	FTD, ALS
Granulin	*GRN*	chr17	FTD
Microtubule-associated protein tau	*MAPT*	chr17	AD, FTD
Apolipoprotein E	*APOE*	chr19	AD
Amyloid-β precursor protein	*APP*	chr21	AD

### Targeted exome sequencing

Genomic DNA from the whole-blood samples was extracted by a commercially available kit (Chemagen, Germany) at the Karolinska institute biobank or by QIAamp DNA Blood Maxi Kit (Qiagen, Germany). All selected exons, including at least 25 nucleotides surrounding the exonic regions, were amplified either using Agilent SureSelect custom design kit or Life Technologies AmpliSeq sequence enrichment method. The amplified DNA was analyzed by Illumina MiSeq or Life Technologies IonTorrent sequencing, respectively. The average target coverage was over 97% for both methods. Targeted exome sequencing was performed at the Uppsala Genome Center using standardized protocols provided by the manufacturer.

### Bioinformatics

Sequenced gene regions were aligned to the human reference genome (assembly hg19). Sequence variations were visualized using Integrated Genome Viewer [49]. Annotation of single nucleotide polymorphisms (SNPs) and small insertions or deletions was performed using Ensembl Variant Effect Predictor (http://grch37.ensembl.org/), NCBI SNP database dbSNP (https://www.ncbi.nlm.nih.gov/snp/), Genome Aggregation Database gnomAD (https://gnomad.broadinstitute.org/) and Alzheimer’s disease mutation database (https://www.alzforum.org/mutations).

## Data Availability

Amyloid-β precursor protein exon 17 nucleotide sequence for the *Uppsala mutation* is available from GenBank (https://www.ncbi.nlm.nih.gov/genbank/, accession number MW892394). Gene based sequencing data summary is available from the corresponding author.

## Abbreviations

AD: Alzheimer’s disease
ALS: Amyotrophic lateral sclerosis
APOE: Apolipoprotein E
APP: Amyloid-β precursor protein
Aβ: Amyloid-β
bvFTD: Behavioral variant of FTD
C9ORF72: C9orf72-SMCR8 complex subunit
CHMP2B: Charged multivesicular body protein 2B
FDG-PET: Fluorodeoxyglucose positron emission tomography
FTD: Frontotemporal dementia
FUS: FUS RNA binding protein
GRN: Granulin
MAPT: Microtubule-associated protein tau
MCI: Mild cognitive impairment
PSEN1: Presenilin 1
PSEN2: Presenilin 2
SNP: Single nucleotide polymorphism
svPPA: Semantic variant of primary progressive aphasia
TARDBP: TDP-43, TAR DNA binding protein
TREM2: Triggering receptor expressed on myeloid cells 2
VCP: Valosin containing protein
